# Supplementary material for: Structure–function analysis of the nsp14 N7–guanine methyltransferase reveals an essential role in Betacoronavirus replication
Source: Proc Natl Acad Sci U S A. 2021 Nov 29;118(49):e2108709118. doi: 10.1073/pnas.2108709118 (PMC8670481; doi:10.1073/pnas.2108709118)
Supplement: Supplementary File [file pnas.2108709118.sapp.pdf]

PNAS
www.pnas.org

Supplementary Information for

Structure-function analysis of the nsp14 N7-guanine methyltransferase reveals an essential role in *Betacoronavirus* replication

Natacha S. Ogando, Priscila El Kazzi, Jessika C. Zevenhoven-Dobbe, Brenda W. Bontes, Alice Decombe, Clara C. Posthuma, Volker Thiel, Bruno Canard, François Ferron, Etienne Decroly & Eric J. Snijder

Eric J. Snijder & Etienne Decroly
Email: E.J.Snijder@lumc.nl & Etienne.decroly@univ-amu.fr

This PDF file includes:

Figures S1 and S2
Tables S1 to S3
SI References

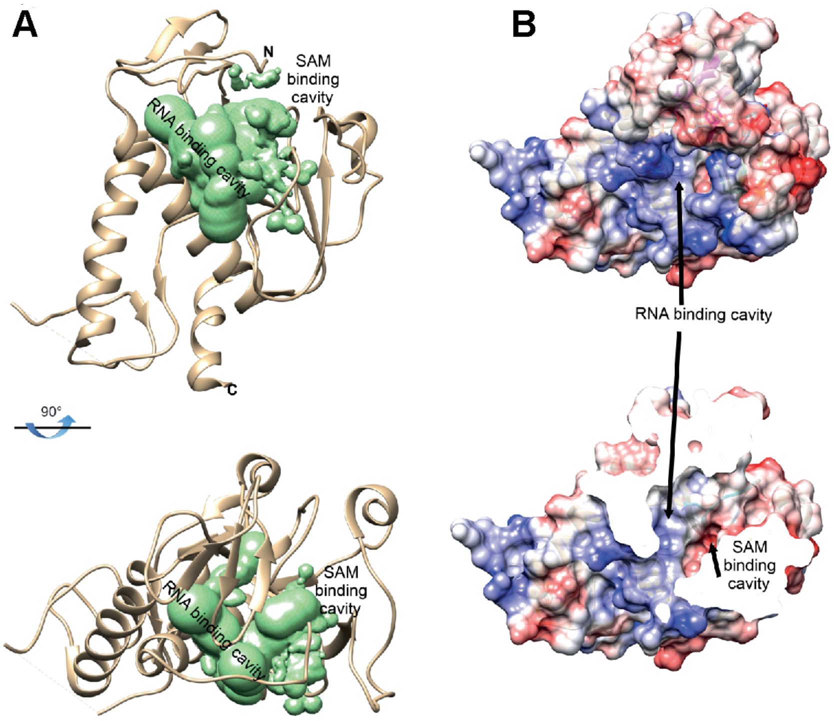

Fig. S1. Structural analysis of the catalytic site and hinge region of the SARS-CoV nsp14 N7-MTase domain. A) Determination of the volume of the enzyme's catalytic site (with the volume depicting the mold of the cavity shown in green). B) Electrostatic surface representation with the surface electrostatic potential calculated by APBS from -10 (red) to +10 (blue) kT/e.

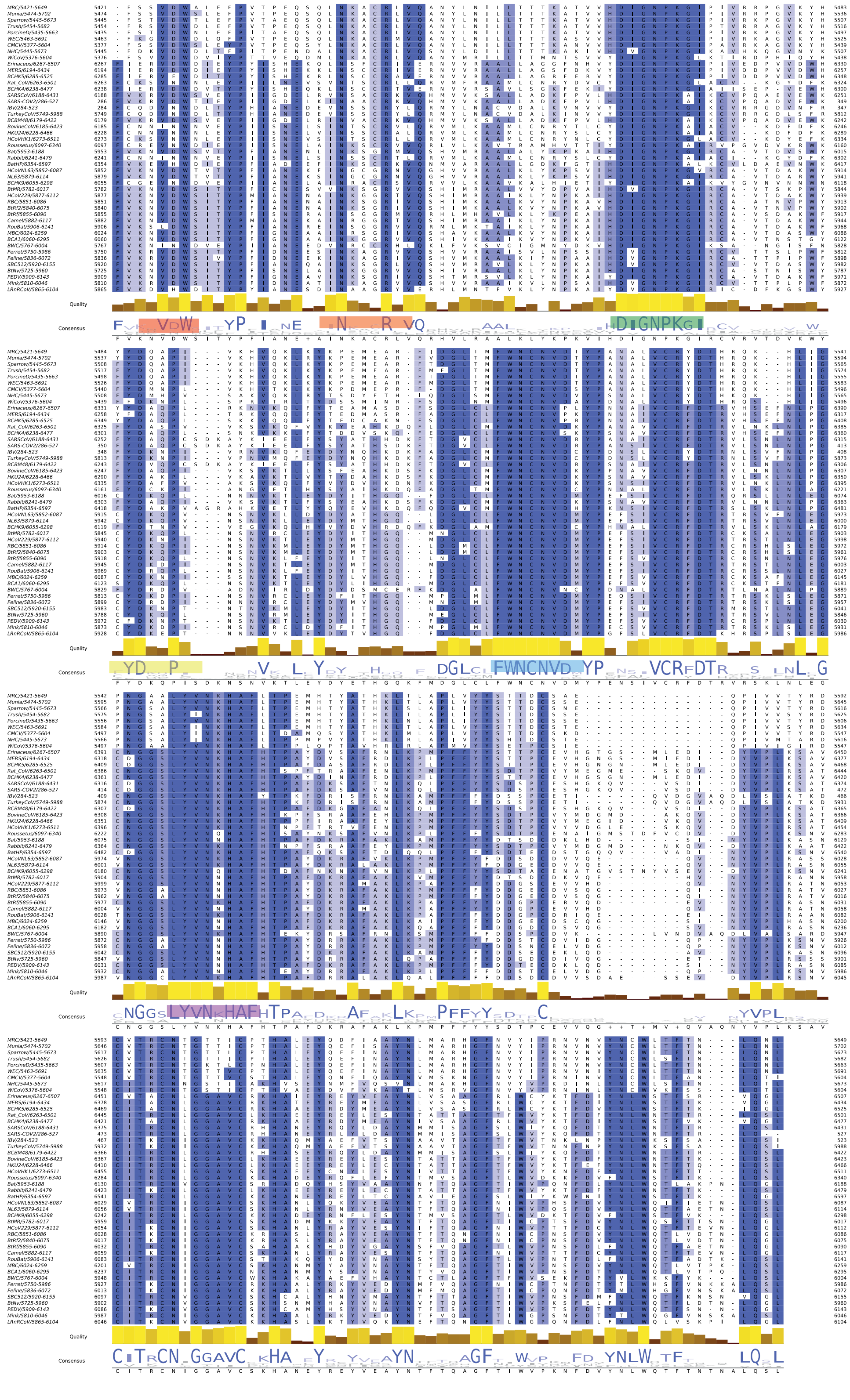

Fig. S2. Alignment of the nsp14 N7-MTase sequences from 47 selected coronaviruses (listed in Table S1). Highly conserved residues are boxed in dark blue (above 70% conservation), while partially conserved residues are displayed in lighter shades of blue. A consensus logo sequence is presented at the bottom of the alignment. The N7-MTase motifs I to VI are highlighted in red, orange, green, yellow, blue, and pink, using the same color scheme as in Fig. 2.

Table S1. List of coronaviruses and NCBI/Brenda-enzymes accession numbers used to extract nsp14 sequences for alignments (Fig. S2) and structural studies.

Virus	Accession number
Alphacoronavirus BtMs-AlphaCoV/GS 2013	A0A0U1WHG4
Avian infectious bronchitis virus (IBV)	P0C6Y2
Bat coronavirus 1A	YP_001718603.1
Bat coronavirus BM48-31	E0XIZ2
Bat coronavirus CDPHE15/USA/200 6	YP_008439224.1
Bat coronavirus HKU4	P0C6W3
Bat coronavirus HKU5	P0C6W4
Bat coronavirus HKU9	P0C6W5
Bat Hp- betacoronavirus	A0A088DIE1
Beluga whale coronavirus SW1	YP_001876435.1
Betacoronavirus Erinaceus	U5KNA9
Betacoronavirus HKU24	A0A0A7UXR0
Bovine coronavirus	P0C6W8
BtMr-AlphaCoV/ SAX2011	A0A0U1UZC3
BtNv-AlphaCoV/SC2013	YP_009201729.1
BtRf-AlphaCoV/HuB2013	YP_009199789.1
Camel alphacoronavirus	ALA50136.1
Common moorhen coronavirus HKU21	H9BR34
Feline infectious peritonitis virus	AGZ84515.1
Ferret coronavirus	YP_009256195.1
Human coronavirus 229E (HCoV-229E)	P0C6X1
Human coronavirus HKU1 (HCoV-HKU1)	P0C6X2
Human coronavirus NL63 (HCoV-NL63)	P0C6X5
Lucheng Rn rat coronavirus	YP_009336483.1
Magpie-robin coronavirus HKU18	H9BR07
Middle East respiratory syndrome-related coronavirus	K9N7C7
Miniopterus bat coronavirus HKU8	YP_001718610.1
Mink coronavirus strain WD1127	YP_009019180
Munia coronavirus HKU13-3514	YP_002308505.1
Murine coronavirus (strain A59) (MHV- A59)	P0C6X9
Night heron coronavirus HKU19	H9BR16
Porcine deltacoronavirus	A0A140ESF0
Porcine epidemic diarrhea virus	NP_839967
Rabbit coronavirus HKU14	H9AA60
Rat coronavirus Parker	YP_009924380.1
Rhinolophus bat coronavirus HKU2	A8JNZ0
Rousettus bat coronavirus	A0A1B3Q5W8
Rousettus bat coronavirus HKU10	AFU92103
Scotophilus bat coronavirus 512	YP_001351683
Severe acute respiratory syndrome coronavirus (SARS-CoV)	P0C6X7
Severe acute respiratory syndrome coronavirus-2 (SARS-CoV-2)	P0DTD1
Sparrow coronavirus HKU17	H9BQZ9
Swine acute diarrhea syndrome related coronavirus BtRf2	AVM80482.1
Thrush coronavirus HKU12	B6VDX7
Turkey coronavirus	YP_001941187
White-eye coronavirus HKU16	YP_005352837.1
Wigeon coronavirus HKU20	H9BR24

Table S2. Projected impact on folding free energy by alanine substitutions of the identified core residues on the SARS-CoV N7-MTase structure, as calculated by PoPMusic (1).

N7-MTase motif	Mutation	Secondary structure*	Solvent accessibility (%)	ΔΔG (kcal/mol)
	**wild-type**	-	-	0
**I**	**W292A**	T	27.44	1.8
**II**	**N306A**	H	39.77	0.72
	**R310A**	H	58.37	0
**III**	**D331A**	H	4.20	0.46
	**K336A**	H	65.79	0.38
**IV**	**D352A**	C	14.21	1.12
**V**	**N386A**	C	32.59	0.99
**VI**	**Y420A**	S	86.78	3.35
	**N422A**	S	10.04	0.92
	**H424A**	S	43.12	2.21
	**F426A**	C	49.59	1.57

*classified as: T, turn; H, α-helix; C, random coil; and S, β-sheet.

Table S3A. Predicted functional impact of individual mutations of key conserved residues in motifs I-III of SARS-CoV, MERS-CoV, and MHV nsp14, predicted with SNAP2 (2).

	SARS-CoV	MERS-CoV	MHV	SARS-CoV	MERS-CoV	MHV	SARS-CoV	MERS-CoV	MHV	SARS-CoV	MERS-CoV	MHV
**aa**	**W292**	**W292**	**W291**	**N306**	**N306**	**N305**	**R310**	**R310**	**R309**	**D331**	**D331**	**D330**
**A**	72	66	78	51	26 *	54	66	30 *	63	75	67	78
**R**	82	81	88	75	32 *	82				91	80	90
**N**	80	63	86				71	41	71	77	78	83
**D**	88	72	91	67	58	78	85	74	84			
**C**	59	62	69	48	22 *	58	65	18 *	63	73	65	76
**Q**	76	74	84	48	35 *	65	60	32 *	61	81	79	84
**E**	82	72	87	73	63	82	81	61	78	70	73	74
**G**	82	82	87	53	41	64	81	31 *	79	79	84	88
**H**	78	62	86	50	36 *	63	59	29 *	60	87	84	89
**I**	72	69	79	71	35 *	77	73	35 *	71	88	85	89
**L**	73	75	82	72	35 *	79	73	21 *	70	90	81	91
**K**	85	84	90	63	59	80	41	20 *	45	91	91	92
**M**	66	71	78	63	34 *	73	66	29 *	65	87	84	89
**F**	45	55	63	74	52	80	81	52	78	89	80	90
**P**	83	89	94	77	64	83	86	70	85	91	91	93
**S**	78	77	78	41	22 *	58	70	32 *	68	74	74	80
**T**	78	80	86	49	24 *	64	69	32 *	66	79	77	83
**W**				81	70	86	87	66	85	92	88	93
**Y**	29 *	50	52	70	54	77	79	53	77	90	83	91
**V**	71	60	78	67	26 *	76	74	38 *	72	87	83	88

SNAP2 scores are predicted with an expected accuracy above 70% confidence, except for scores labeled with *, which are predicted with an expected accuracy above 53% confidence. Positive values indicate a destabilizing effect, while negative values indicate a neutral effect.

Table S3B. Predicted functional impact of individual mutations of key conserved residues in motifs III-VI of SARS-CoV, MERS-CoV, and MHV nsp14, predicted with SNAP2 (2).

	**SARS-CoV**	**MERS-CoV**	**MHV**	**SARS-CoV**	**MERS-CoV**	**MHV**	**SARS-CoV**	**MERS-CoV**	**MHV**	**SARS-CoV**	**MERS-CoV**	**MHV**
**aa**	**K336**	**K336**	**K335**	**D352**	**D352**	**D349**	**N386**	**N382**	**N380**	**Y420**	**Y416**	**Y414**
**A**	31 *	20 *	39 *	34 *	63	73	60	45	56	71	66	74
**R**	-17 *	-20 *	-1 *	45	85	87	78	73	77	89	85	91
**N**	28 *	26 *	44	22 *	73	69				86	71	88
**D**	69	63	76				62	56	63	90	73	92
**C**	22 *	7 *	-16 *	27 *	57	69	60	15	57	61	54	64
**Q**	16 *	6 *	29 *	43	74	73	47	52	64	82	77	86
**E**	52	45	60	26 *	67	53	75	70	74	87	82	89
**G**	52	41	60	49	77	78	51	55	49	87	81	88
**H**	7 *	-6 *	19 *	21 *	74	80	63	51	63	59	70	78
**I**	-15 *	-70	-40 *	59	78	86	77	53	76	69	62	70
**L**	22 *	8 *	33 *	61	80	88	80	49	78	74	70	74
**K**				48	87	88	76	71	76	90	86	91
**M**	10 *	-39 *	18 *	55	78	84	74	52	73	78	72	79
**F**	52	41	55	23 *	71	87	81	67	80	42	47	40
**P**	62	30 *	69	72	89	89	71	76	82	88	91	94
**S**	22 *	13 *	36 *	29 *	65	65	44	31	42	85	69	86
**T**	26 *	13 *	39 *	34 *	69	69	51	30 *	33 *	86	79	88
**W**	67	56	67	66	81	90	87	78 *	86	68	71	71
**Y**	48	35 *	51	32 *	76	86	79	38 *	78			
**V**	22 *	8 *	32 *	53	76	83	75	41	75	68	49	70

SNAP2 scores are predicted with an expected accuracy above 70% confidence, except for scores labeled with *, which are predicted with an expected accuracy above 53% confidence. Positive values indicate a destabilizing effect, while negative values indicate a neutral effect.

Table S3C. Predicted functional impact of individual mutations of key conserved residues in motif VI of SARS-CoV, MERS-CoV, and MHV nsp14, predicted with SNAP2 (2).

	**SARS-CoV**	**MERS-CoV**	**MHV**	**SARS-CoV**	**MERS-CoV**	**MHV**	**SARS-CoV**	**MERS-CoV**	**MHV**
**aa**	**N422**	**N418**	**N416**	**H424**	**H420**	**H418**	**F426**	**F422**	**F420**
**A**	66	33 *	69	63	62	64	56	59	67
**R**	81	72	84	74	26 *	35 *	83	81	85
**N**				63	70	65	82	79	83
**D**	64	65	79	84	86 *	85	89	88	90
**C**	61	41	66	64	20	64	48 *	39 *	50
**Q**	64	4 *	70	65	69	67	78	72	80
**E**	80	71	83	79	81	80	84	72	85
**G**	64	20 *	64	75	66	76	81	79	83
**H**	60	49	67				59	76	79
**I**	79	71	83	76	69	76	58 *	10 *	60
**L**	81	72	85	76	41	75	63	50	59
**K**	79	69	81	81	81	82	85	82	87
**M**	76	63	80	72	65	72	60	49	47
**F**	69	74	85	64	75	76			
**P**	84	76	87	90	90	90	89	87	91
**S**	52	-3 *	59	70	71	72	79	75	80
**T**	59	42	65	64	72	76	78	60	80
**W**	88	81	90	81	82	82	69	69	67
**Y**	79	71	83	65	70	67	49	49	49
**V**	78	65	81	71	54	72	62 *	37 *	65

SNAP2 scores are predicted with an expected accuracy above 70% confidence, except for scores labeled with *, which are predicted with an expected accuracy above 53% confidence. Positive values indicate a destabilizing effect, while negative values indicate a neutral effect.

SI References

1. Y. Dehouck, J. M. Kwasigroch, D. Gilis, M. Rooman, PoPMuSiC 2.1: a web server for the estimation of protein stability changes upon mutation and sequence optimality. *BMC Bioinformatics* **12**, 151 (2011).
2. M. Hecht, Y. Bromberg, B. Rost, Better prediction of functional effects for sequence variants. *BMC Genomics* **16 Suppl 8**, S1 (2015).
